# Multiplex PCR assay to detect high risk lineages of *Salmonella* Typhi and Paratyphi A

**DOI:** 10.1371/journal.pone.0267805

**Published:** 2022-07-22

**Authors:** Fahad Khokhar, Derek Pickard, Zoe Dyson, Junaid Iqbal, Agila Pragasam, Jobin Jacob John, Balaji Veeraraghavan, Farah Qamar, Gordon Dougan, Hilary MacQueen, Sushila Rigas, Mark Holmes, Ankur Mutreja

**Affiliations:** 1 Department of Medicine, Cambridge Institute of Therapeutic Immunology & Infectious Disease (CITIID), University of Cambridge, Cambridge, United Kingdom; 2 London School of Hygiene & Tropical Medicine, London, United Kingdom; 3 Department of Infectious Diseases, Central Clinical School, Monash University, Melbourne, Victoria, Australia; 4 Wellcome Sanger Institute, Wellcome Genome Campus, Hinxton, Cambridge, United Kingdom; 5 Department of Paediatrics and Child Health, Aga Khan University, Karachi, Pakistan; 6 Department of Clinical Microbiology, Christian Medical College, Vellore, Tamil Nadu, India; 7 The Wellcome Trust, London, United Kingdom; 8 School of Life, Health & Chemical Sciences, The Open University, Milton Keynes, United Kingdom; 9 Department of Veterinary Medicine, University of Cambridge, Cambridge, United Kingdom; Manipal College of Medical Sciences, NEPAL

## Abstract

Enteric fever infections remain a significant public health issue, with up to 20 million infections per year. Increasing rates of antibiotic resistant strains have rendered many first-line antibiotics potentially ineffective. Genotype 4.3.1 (H58) is the main circulating lineage of *S*. Typhi in many South Asian countries and is associated with high levels of antibiotic resistance. The emergence and spread of extensively drug resistant (XDR) typhoid strains has increased the need for a rapid molecular test to identify and track these high-risk lineages for surveillance and vaccine prioritisation. Current methods require samples to be cultured for several days, followed by DNA extraction and sequencing to determine the specific lineage. We designed and evaluated the performance of a new multiplex PCR assay, targeting *S*. Paratyphi A as well as the H58 and XDR lineages of *S*. Typhi on a collection of bacterial strains. Our assay was 100% specific for the identification of lineage specific *S*. Typhi and *S*. Paratyphi A, when tested with a mix of non-Typhi *Salmonella* and non-*Salmonella* strains. With additional testing on clinical and environmental samples, this assay will allow rapid lineage level detection of typhoid of clinical significance, at a significantly lower cost to whole-genome sequencing. To our knowledge, this is the first report of a SNP-based multiplex PCR assay for the detection of lineage specific serovars of *Salmonella* Typhi.

## Introduction

Enteric fever is caused by the bacteria *Salmonella enterica* serovars Typhi and Paratyphi A, B and C. It remains a significant health issue, which is estimated to cause up to 20 million infections and 161,000 deaths per year, predominantly in low and middle-income countries in Southeast Asia and Africa [1]. *S*. Typhi and *S*. Paratyphi A are human restricted pathogens and are transmitted by the faecal-oral route often via contaminated water [1]. Vaccination, access to clean water, and improved sanitation are effective means to prevent transmission of typhoid.

Cases of typhoid fever can be treated with former first-line antibiotics such as chloramphenicol, ampicillin or co-trimoxazole. However, the emergence of multi-drug resistant (MDR) *S*. Typhi in the mid-1970s and the recent emergence of strains with increased anti-microbial resistance (AMR) profiles to more antibiotics [2], has rendered these treatment options potentially ineffective in the near future. Many MDR *S*. Typhi strains possess self-transmissible incompatibility type (IncHI1) plasmids carrying a suite of antimicrobial resistance genes [2, 3]. Haplotype 58 (H58) or genotype 4.3.1 is the dominant *S*. Typhi lineage in many parts of Asia and Eastern Africa today and is associated with high levels of multidrug resistance and reduced susceptibility to fluoroquinolones [2].

Extensively drug-resistant (XDR) *S*. Typhi, first reported in Pakistan in 2016 [3], was found to be an MDR H58 strain that had acquired an IncY plasmid from an *E*. *coli* isolate harbouring both *bla*CTX-M-15 and *qnrS* resistance genes conferring resistance to ceftriaxone and third generation cephalosporins. This leaves azithromycin as the only viable oral treatment option for XDR *S*. Typhi. However, recent reports of azithromycin-resistant *S*. Typhi and *S*. Paratyphi A in Bangladesh [4] and Pakistan [5] have raised concerns about the potential of this mutation to evolve in XDR strains in the future, further limiting treatment options.

Current laboratory diagnosis of typhoid fever requires clinical samples to be sent to a centralised laboratory to be processed by bacterial culture and standard identification, followed by susceptibility, serological and/or advanced molecular tests such as real-time PCR and whole-genome sequencing (WGS). Blood culture remains the diagnostic technique of choice but takes several days for results and only identifies 45–70% of confirmed cases. It is also limited by the low numbers of *Salmonella* bacteria in samples ranging from <1–22 organisms/ml of blood [6, 7]. Sampling from bone marrow has been shown to have greater sensitivity and specificity but that remains a highly invasive procedure. There have been several studies that utilise PCR assays to identify invasive *Salmonella* serovars [8–14] but rely on blood culture over 2–3 days followed by DNA extraction before PCR testing. More recently, studies have shown that the use of ox bile-containing media for the enrichment of bacteria directly from blood samples [15], or the selective removal of human DNA [16], combined with a PCR assay can reduce the turnaround time for diagnosis and increase the diagnostic sensitivity [17]. Most importantly, however, is that none of the current routine diagnostic platforms highlighted here offer a resolution to discriminate between low and high-risk lineages in a single test.

In our study, we designed a simple, rapid, highly specific multiplex PCR assay for the detection of *S*. Typhi, *S*. Paratyphi A, *S*. Typhi H58 and *S*. Typhi XDR, based on single nucleotide polymorphisms (SNPs) specific for these lineages of clinical significance. Once tested on clinical and environmental samples, this will be used to guide clinical and public health decision making.

## Methods

### DNA extraction and bacterial strains

A total of 107 bacterial isolates were used in this study. All non-XDR isolates in Table 1 were cultured onto Luria-Bertani (LB) agar plates from frozen stock vials. A single bacterial colony was picked to subculture into 1 ml of LB broth and incubated overnight at 37°C in a shaking incubator. Genomic DNA was extracted from overnight cultures using the Wizard Genomic DNA Purification Kit (Promega, USA). Genomic DNA extracted from five *S*. Typhi XDR strains was supplied by Aga Khan University, Karachi, Pakistan. The additional 75 samples that were obtained for screening using our PCR assay, had been previously identified by Matrix-assisted laser desorption/ionization time-of-flight mass spectrometry (MALDI-TOF MS) as *S*. Typhi or *S*. Paratyphi A, DNA extracted, but not whole-genome sequenced. All DNA strains used were quantified using the Qubit 3 fluorometer with the dsDNA broad-range assay kit (Thermo Fisher Scientific, UK) and diluted in 10 mM Tris buffer.

**Table 1:** Bacterial DNA strains used in testing. All *Salmonella* strains are serovars of *subspecies enterica* unless otherwise stated.

Bacterial strain	Isolate details	Country/Year	Accession Number
*S*. Typhi BRD948	H10/15 Ty2 (Δ*aroC aroD htrA)*	Russia/1918	AE014613
*S*. Typhi SGB87	98–0664 (H55)	Nepal/2006	GCA_001362215.1
*S*. Typhi	Quail strain	USA/1958	NA
*S*. Typhi	lupe GEN0059 (4.1)	Samoa/2012	NA
*S*. Typhi	403 Ty (H59 3.1.2)	Indonesia/2002	NA
*S*. Paratyphi A	AKU_12601	Pakistan/2002	FM200053
*S*. Paratyphi B	SPB7	USA	NC_010102
*S*. Typhi H58	ERL12148	India/2012	LT883153.1
*S*. Typhi H58	ERL12960	India/2012	LT904894
*S*. Typhi XDR 1	-	Pakistan	NA
*S*. Typhi XDR 2	-	Pakistan	NA
*S*. Typhi XDR 3	-	Pakistan	NA
*S*. Typhi XDR 4	-	Pakistan	NA
*S*. Typhi XDR 5	-	Pakistan	NA
*S*. Enteritidis	PT4/NCTC 13349	UK/2004	AM933173
*S*. Hadar	SC1	UK/1996	NA
*S*. Infantis	SC31	UK	NA
*S*. Cholearasuis	SC-B67	USA/2002	AE017220
*S*. *enterica subsp*. *arizonae*	CDC 346–86		CP000880.1
*S*. *enterica subsp*. *diarizonae*	CDC 01–0005	USA/2001	NA
*S*. Dublin	TYT3627	-	AY14490
*S*. Newport	E2002001708	-	ABEW01000000
*S*. 1,4, (5),12:i	CVM23701	-	See below
*S*. Kentucky	CVM29188	-	NA
*S*. Heidelberg	CVM30485	-	CP001120.1
*S*. Javiana	CVM35943	-	NA
*S*. Saint Paul	SARA29	-	ABAN01000001-ABAN01000182
*S*. Pullorum	S449/87		CP000857.1
*S*. Typhimurium	D23580	Malawi/2004	FN424405.1
*S*. Typhimurium	ST4/74	UK/1966	CP002487.1
*Esherichia coli*	BL21	-	-
*Vibrio cholerae*	O1 El Tor	-	-

### Primer design

Primers targeting a highly conserved gene and an intergenic region were designed for the detection of *S*. Typhi and *S*. Paratyphi A serovars respectively (Table 2). Primers with additional mutations incorporated based on the mismatch amplification mutation assay (MAMA) PCR principle as previously described [18], were designed to selectively amplify a target sequence in the presence of a SNP of interest for H58 and XDR lineages [19]. All primers were designed using Primer-BLAST [20], synthesised by Integrated DNA Technologies (IDT, USA) and were resuspended from their lyophilised form in 10 mM Tris buffer. Appropriate amplicon lengths were selected for each target with a minimum of 50 base pairs (bp) difference, in order to effectively separate and be visualised by gel electrophoresis.

**Table 2:** Primer sequences used in our multiplex assay with expected amplicon sizes generated for each target.

Target	Primer name	Gene Target	Primer sequence (5’-3’)	Amplicon length (bp)
*S*. Typhi	ST_227F	STY0307	GGCAGATATACTTTCGCAGGCA	227
	ST_227R		CCCAGAACCAAATTTGCTTACA	
*S*. Paratyphi A	SPAI_305F	Intergenic region SSPA1732a –SSPA1724	CGCAGAGTGCAAGTGGAGT	305
	SPAI_305R		GCATCCTCGGCCAGTCTTAC	
*S*. Typhi XDR	XDR_425F	STY0962	TGAATGGTTCTGGTCTGGCG	425
	XDR_425R		CTAAACCACGACGGCTCAGT	
*S*. Typhi H58	H58_509F	STY2513	GGGCTTGATGGCTTCATTAGT	509
	H58_509R		ACAGGTTGTACGCCTTTCCA	

### PCR protocol

Each PCR reaction contained 5 ng of DNA, 12.5 μl of 2X PCR Master Mix (Thermo Fischer Scientific, UK), 4, 8 or 10 μM each of the forward and reverse primers (Table 2), and nuclease free water to a final volume of 25 μl. All reactions were performed on a T100 thermal cycler (BioRad Laboratories Inc., USA) under the following cycling conditions; 95°C for 2 mins, followed by 30 cycles of 95°C for 30 sec, 60°C for 45 sec, 68°C for 1 min, and final extension at 68°C for 10 mins. The PCR products were run on 1.2% (w/v) agarose gels containing SYBR^®^ Safe DNA Gel Stain (Invitrogen, USA) and visualised on a ChemiDoc MP imaging system (BioRad Laboratories Inc., USA).

## Results

**Fig 1:**
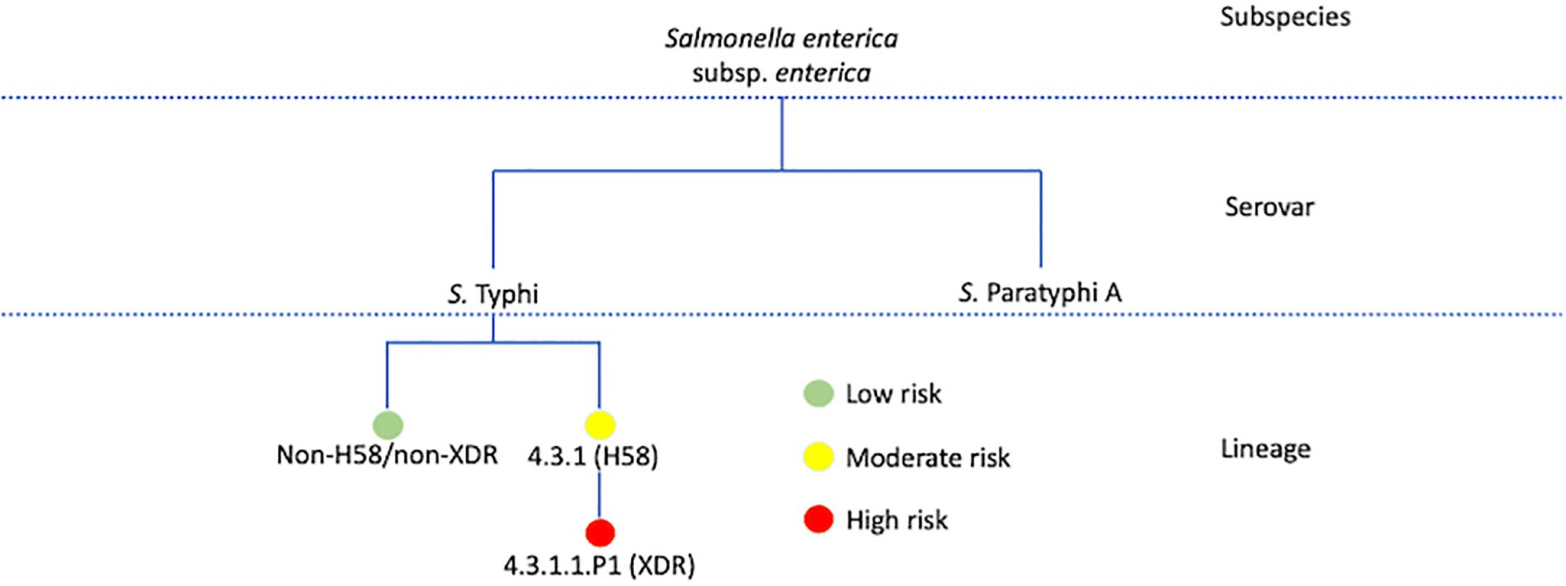
The developed SNP-based multiplex PCR assay detects *Salmonella* species and offers greater resolution down to lineage specific level. Current routine diagnostics can only offer a resolution to species or serovar level. From the data obtained through whole genome sequencing, our targets for this PCR assay can identify specific lineages of *S*. Typhi that can be of clinical importance.

Our multiplex assay not only distinguished *S*. Paratyphi A from *S*. Typhi, but also identified low and high-risk lineages of *S*. Typhi that are of clinical relevance (Fig 1). Optimal conditions were initially determined for singleplex PCR, before adapting for the multiplex reaction. The primers were designed from specific regions in their reference genomes that are highly conserved across all strains, genes STY0307 for *S*. Typhi. and an intergenic region between SSPA1723a and SSPA1724 for *S*. Paratyphi A. The *S*. Typhi and *S*. Paratyphi A specific primers yielded 227 bp and 305 bp amplicon products, respectively.

Previous studies have screened thousands of publicly available *Salmonella* genomes and have identified SNPs that are specific for certain lineages [19, 21]. These were chosen for use as relevant diagnostic markers for our PCR assay. Our marker for the multidrug resistant lineage 4.3.1 (H58) is based on a C→T synonymous mutation (T349T) in the STY2513 gene at position 2348902 in the *S*. Typhi CT18 reference genome, which encodes for the anaerobic glycerol-3-phosphate dehydrogenase subunit A (glpA) gene. This mutation covers all H58 4.3.1 lineage and sub-lineage isolates, with our primers generating a 509 bp sized amplicon product. The diagnostic marker for the XDR lineage (genotype 4.3.1.1.P1) is based on a G→A (E13E) synonymous mutation in the STY0962 gene encoding anaerobic dimethyl sulfoxide reductase chain A precursor (dmsA), at position 955875 of the CT18 genome. Our XDR primers targeting this SNP generate an amplicon size of 425 bp. Our results showed that our initial primers designed for the H58 and XDR targets with just the one SNP incorporated were not specific for these lineages. S1 Fig shows the results for our initial one SNP XDR primers, showing amplification when using non-XDR DNA samples (lanes 1–5). Further adaptation was made by incorporating an additional SNP at the penultimate base at the 3’ end of the primer, which resulted in no amplification in non-XDR DNA samples (lanes 7–24). Note that these original XDR primers generated a 369 bp amplicon size which was later modified (425 bp) to incorporate other targets into the multiplex assay.

**Fig 2:**
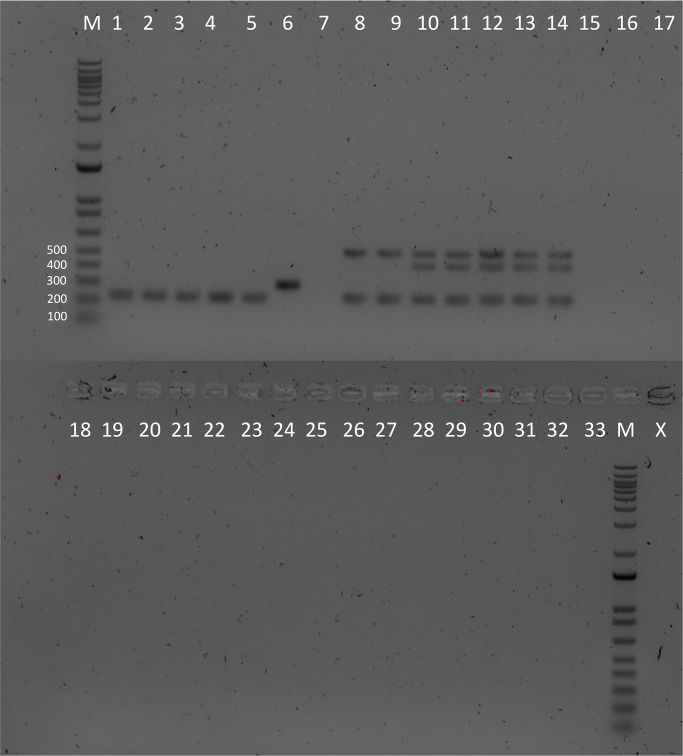
Pairwise alignment of the CT18 reference genome against an H58 (A) and XDR (B) genome showing the nucleotide position along the top and SNPs identified for our custom primer design highlighted underneath. The SNP of interest is highlighted in green in the H58 and XDR primer sequences, with the additional added mutation highlighted in yellow. The addition of a second mutation induced the specificity of the primers to bind to only our target. Sequences are shown in 5’-3’ direction unless otherwise indicated. Alignments produced and visualised using Geneious Prime v2021.1.1.

**Fig 3:**
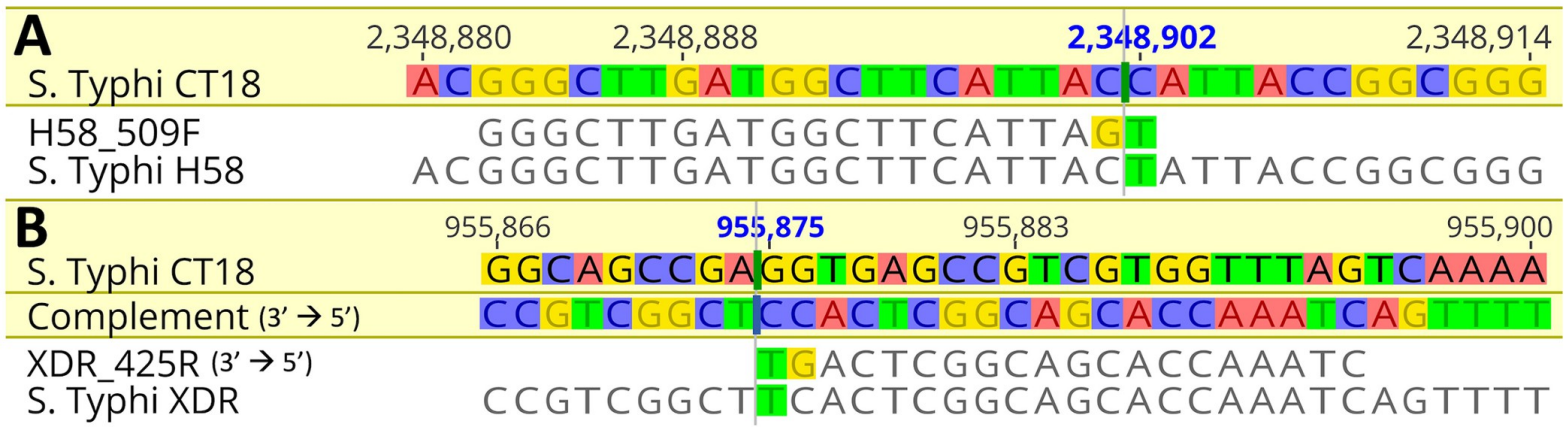
Gel image showing the results of our multiplex PCR assay on a range of non-Typhi *Salmonella* and non-*Salmonella* DNA samples. Results show no positive reaction with non-Typhi serotypes or non-*Salmonella* samples, with all expected amplicon bands present in the target samples. Lane M, 1 kb DNA ladder; lane 1, *S*. Typhi BRD948; lane 2, *S*. Typhi SGB87; lane 3 *S*. Typhi Quail strain; lane 4, *S*. Typhi lupe GEN0059; lane 5, *S*. Typhi 403 Ty; lane 6, *S*. Paratyphi A; lane 7, *S*. Paratyphi B; lane 8, *S*. Typhi H58 12148; lane 9, *S*. Typhi H58 12960; lanes 10–14, *S*. Typhi XDR1-5; lane 15, *S*. Enteritidis; lane 16, *S*. Hadar; lane 17, *S*. Infantis; lane 18, *S*. Cholerasuis; lane 19, *S*. enterica subsp. arizonae; lane 20, *S*. enterica subsp. diarizonae; lane 21, *S*. Dublin; lane 22, *S*. Newport; lane 23, *S*. 1,4, (5),12:i; lane 24, *S*. Kentucky; lane 25, *S*. Heidelberg; lane 26, *S*. Javiana; lane 27, *S*. Saint Paul; lane 28, *S*. Pullorum; lane 29, *S*. Typhimurium D23580; lane 30, *S*. Typhimurium 4/74; lane 31, *E*. *coli* BL21; lane 32, *V*. *cholerae* O1 El Tor; lane 33, no template control (NTC); lane X, not used.

Fig 2 shows the pairwise alignment of the final H58 and XDR primers against the CT18 reference genome, showing the incorporation of both the SNP of interest and an additional mutation. To determine the specificity of the assay, we tested the multiplex primers on a range of non-Typhi *Salmonella* and non-*Salmonella* pathogens. The additional mutation in the H58 and XDR primers induced the specificity for the target sequence and strain and subsequently produced no false positive results (Fig 3).

From the additional DNA samples obtained for testing, 74/75 were originally identified as *S*. Typhi, with the remaining isolate identified as *S*. Paratyphi A by MALDI-TOF MS. Using the same PCR conditions listed above, we tested our assay on these samples, with the single *S*. Paratyphi A sample confirmed by producing only a single 305 bp band as expected. Interestingly, 13/74 isolates that had previously been identified as *S*. Typhi, also only produced the 305 bp *S*. Paratyphi A band in our assay. To investigate these discrepant results, these isolates were later whole-genome sequenced and confirmed as *S*. Paratyphi A. Of the remaining isolates, our PCR assay identified 53/75 as *S*. Typhi H58, 4/75 as *S*. Typhi non-H58/non-XDR, 1/75 as mixed *S*. Typhi and *S*. Paratyphi A, and 3/75 did not produce any bands (S1 Table). None of these additional 75 DNA isolates were identified as *S*. Typhi XDR.

## Discussion

Typhoid fever remains a major public health issue particularly in South and Southeast Asia. Early diagnosis is important for detecting cases in patients but also to discover sources of potential outbreaks. It is widely accepted that improved methods for the diagnosis and monitoring of the emergence and spread of *S*. Typhi would facilitate disease control and treatment. Yet there remain difficulties in obtaining sufficient samples for rapid molecular testing as direct blood samples are limited by the low abundance of *Salmonella* bacteria and other sampling methods remain extremely invasive and not widely available. Standard diagnostic techniques also lack the resolution to discriminate between different serovars of *S*. Typhi as well as drug-susceptible from drug-resistance strains. An ideal diagnostic for typhoid fever should be high-resolution, rapid, specific, and sensitive for the target organism.

The widespread dissemination of the H58 lineage in multiple countries, XDR lineage across Pakistan, and the emergence of azithromycin resistant isolates is of concern. The only method to identify and track the spread of such resistant strains is by whole genome sequencing. Based on our SNP-based diagnostic assay, there is potential to design and incorporate PCR primers as an additional target to monitor the spread of such strains at a significantly lower cost compared to complete genome sequencing.

Our multiplex PCR assay for the detection of *S*. Typhi and *S*. Paratyphi A enables the identification of the MDR H58 and XDR *S*. Typhi lineages. In many countries, *S*. Paratyphi A infections are increasingly common, so it was important that this assay could distinguish its presence in a population [22]. Our single and multiplex PCR assays found no false positive reaction with non-Typhi serotypes or non-*Salmonella* pathogens, suggesting that the target genes are specific for our *Salmonella* serovar targets. From the additional DNA samples obtained, our assay was able to correctly identify *S*. Paratyphi A isolates that had previously been classified as *S*. Typhi by MALDI-TOF MS, which were subsequently confirmed as *S*. Paratyphi A by whole-genome sequencing. Although our assay was able to positively identify XDR *S*. Typhi from extracted DNA from five samples, we acknowledge there is a lack of additional XDR isolates in our collection. A further limitation is that our experiments were performed using DNA extracted from purified cultures and therefore contained few potential PCR inhibitors. Further optimisation of our PCR assay is planned for working directly on bacterial colonies in addition to sampling from environmental water and sewage samples. As direct assays are developed for clinical use in the future, our PCR assay should directly plug in to obtain the lineage level resolution.

We envision that our multiplex PCR assay will be widely used in routine laboratory diagnosis from DNA extracted after blood culture incubation, and eventually in combination with previously mentioned enrichment methods. Having a low cost, high-resolution, simple PCR test, as opposed to the current expensive whole-genome sequencing methods, allows our assay to be accessible to low- and middle-income countries where improved diagnostics for typhoid fever are most needed. The lineage level rapid detection of *S*. Typhi will make clinical decision making more efficient and help tackle AMR.

## Supporting information

S1 Fig Gel image showing the results of our singleplex PCR assay using one SNP and multi-SNP *S*. Typhi XDR Primers on non-XDR DNA samples. Results show that reactions using XDR primers with just the one SNP show amplification on non-XDR DNA samples. However, with the addition of a second SNP at the 3’ end of the primer, no amplification is present in the same non-XDR DNA samples. Lane M, 1kb DNA ladder; lanes 1, 7, 13 and 19 *S*. Typhi BRD948; lanes 2, 8, 14 and 20; *S*. Paratyphi A; lanes 3, 9, 15 and 21, *S*. Paratyphi B; lanes 4, 10, 16 and 22, *S*. Typhi H58 12148; lanes 5, 11, 17 and 23, *S*. Typhi H58 12960; lanes 6, 12, 18 and 24, No template control (NTC).
(PNG)

S1 Table Summary of results from testing our multiplex assay on the additional 75 DNA samples plus controls. Samples identified as *S*. Paratyphi A with our PCR assay are highlighted in green. For these samples the result of subsequent result of whole genome sequencing is also listed. Samples that did not produce any bands are highlighted in yellow.
(DOCX)

S1 Raw images (PDF)
