# Correction: Multiplex PCR assay to detect high risk lineages of *Salmonella* Typhi and Paratyphi A

**DOI:** 10.1371/journal.pone.0362150

**Published:** 2026-11-05

**Authors:** Fahad Khokhar, Derek Pickard, Zoe Dyson, Junaid Iqbal, Agila Pragasam, Jobin Jacob John, Balaji Veeraraghavan, Farah Qamar, Gordon Dougan, Hilary MacQueen, Sushila Rigas, Mark Holmes, Ankur Mutreja

Following the publication of this article [1], concerns were raised regarding Figs 2 and 3. Specifically, Figs 2 and 3 do not appear to match their corresponding captions.

The second author stated that Figs 2 and 3 appear in the incorrect order in [1], that the gel image in Fig 2 in [1] should be Fig 3, and that the primer sequences in Fig 3 in [1] should be Fig 2. Please see the corrected figures below.

The first author also stated that the gel image in Fig 2 in [1] is incorrect. The correct version of this figure is provided here as Fig 3. The underlying image for the corrected figure is provided in S1 Raw images in [1].

**Fig 2:**
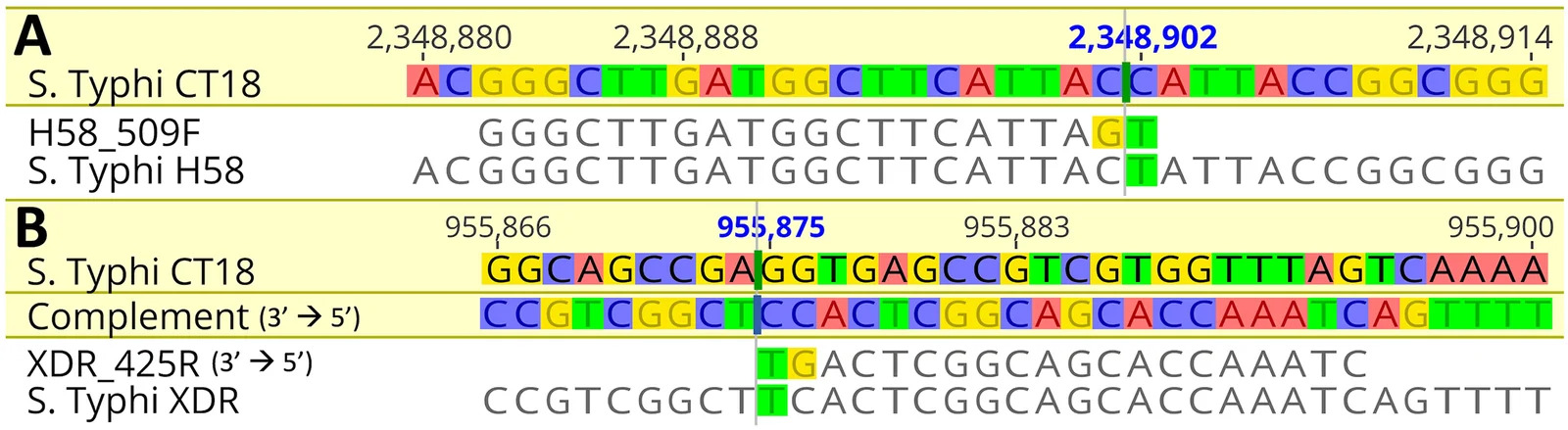
Pairwise alignment of the CT18 reference genome against an H58 (A) and XDR (B) genome showing the nucleotide position along the top and SNPs identified for our custom primer design highlighted underneath. The SNP of interest is highlighted in green in the H58 and XDR primer sequences, with the additional added mutation highlighted in yellow. The addition of a second mutation induced the specificity of the primers to bind to only our target. Sequences are shown in 5’-3’ direction unless otherwise indicated. Alignments produced and visualised using Geneious Prime v2021.1.1.

**Fig 3:**
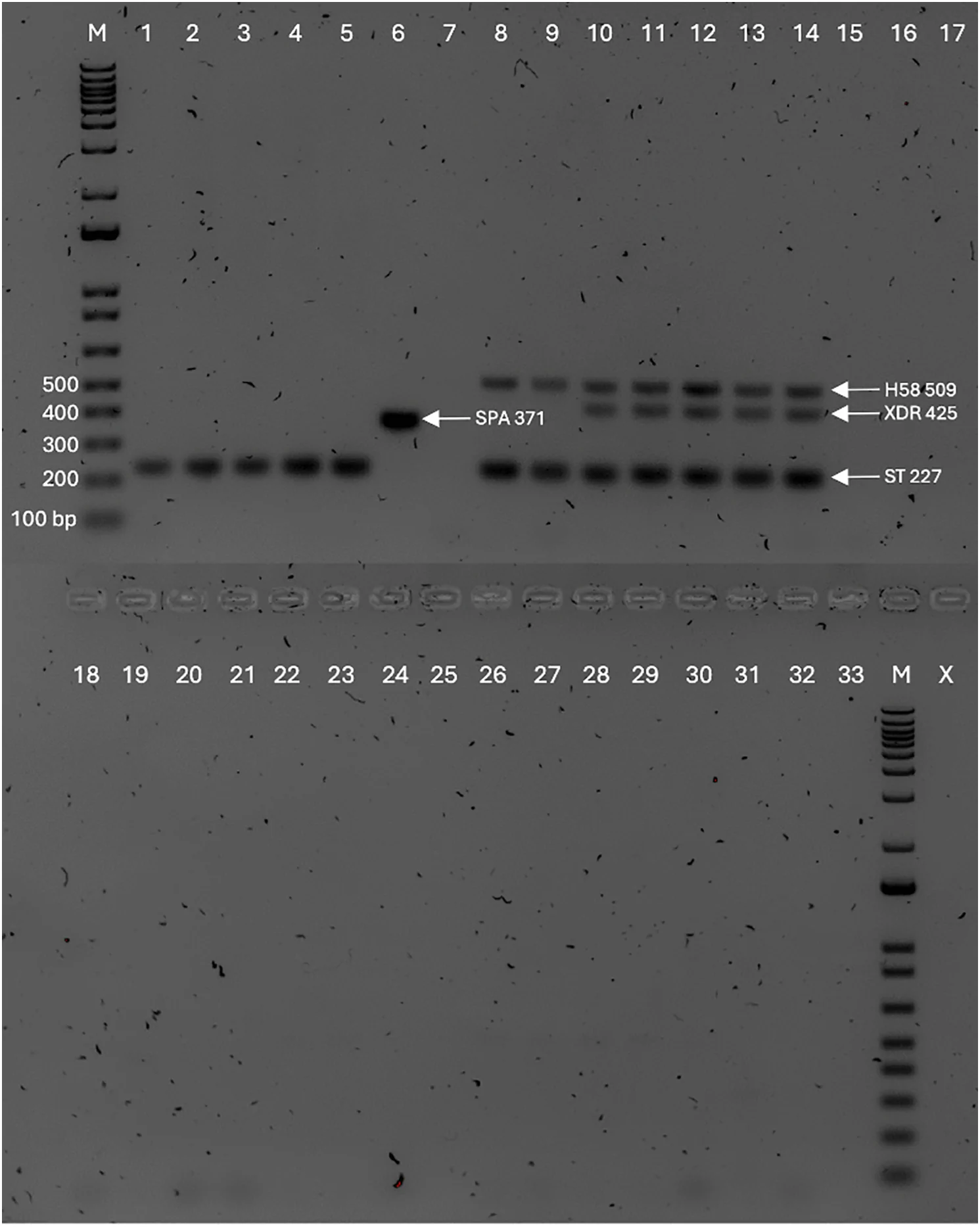
Gel image showing the results of our multiplex PCR assay on a range of non-Typhi *Salmonella* and non-*Salmonella* DNA samples. Results show no positive reaction with non-Typhi serotypes or non-*Salmonella* samples, with all expected amplicon bands present in the target samples. Lane M, 1 kb DNAladder; lane 1, *S.* Typhi BRD948; lane 2, *S.* Typhi SGB87; lane 3 *S.* Typhi Quail strain; lane 4, *S.* Typhi lupe GEN0059; lane 5, *S.* Typhi 403 Ty; lane 6, *S.* Paratyphi A; lane 7, *S.* Paratyphi B; lane 8, *S.* Typhi H58 12148; lane 9, *S.* Typhi H58 12960; lanes 10–14, *S.* Typhi XDR1-5; lane 15, *S.* Enteritidis; lane 16, *S.* Hadar; lane 17, *S.* Infantis; lane 18, *S.* Cholerasuis; lane 19, *S.* enterica subsp. arizonae; lane 20, *S.* enterica subsp. diarizonae; lane 21, *S.* Dublin; lane 22, *S.* Newport; lane 23, *S.* 1,4, (5),12:i; lane 24, *S.* Kentucky; lane 25, *S.* Heidelberg; lane 26, *S.* Javiana; lane 27, *S.* Saint Paul; lane 28, *S.* Pullorum; lane 29, *S.* Typhimurium D23580; lane 30, *S.* Typhimurium 4/74; lane 31, *E. coli* BL21; lane 32, *V. cholerae* O1 El Tor; lane 33, no template control (NTC); lane X, not used.

## References

[1] Khokhar F, Pickard D, Dyson Z, Iqbal J, Pragasam A, John JJ, et al. Multiplex PCR assay to detect high risk lineages of *Salmonella* Typhi and Paratyphi A. PLoS One. 2022;17(7):e0267805. doi: 10.1371/journal.pone.0267805 35867662 PMC9307194

