# Supplementary figures and images for: Multiplex PCR assay to detect high risk lineages of Salmonella Typhi and Paratyphi A
Source: PLoS One. 2022 Jul 22;17(7):e0267805. doi: 10.1371/journal.pone.0267805 (PMC9307194; doi:10.1371/journal.pone.0267805)

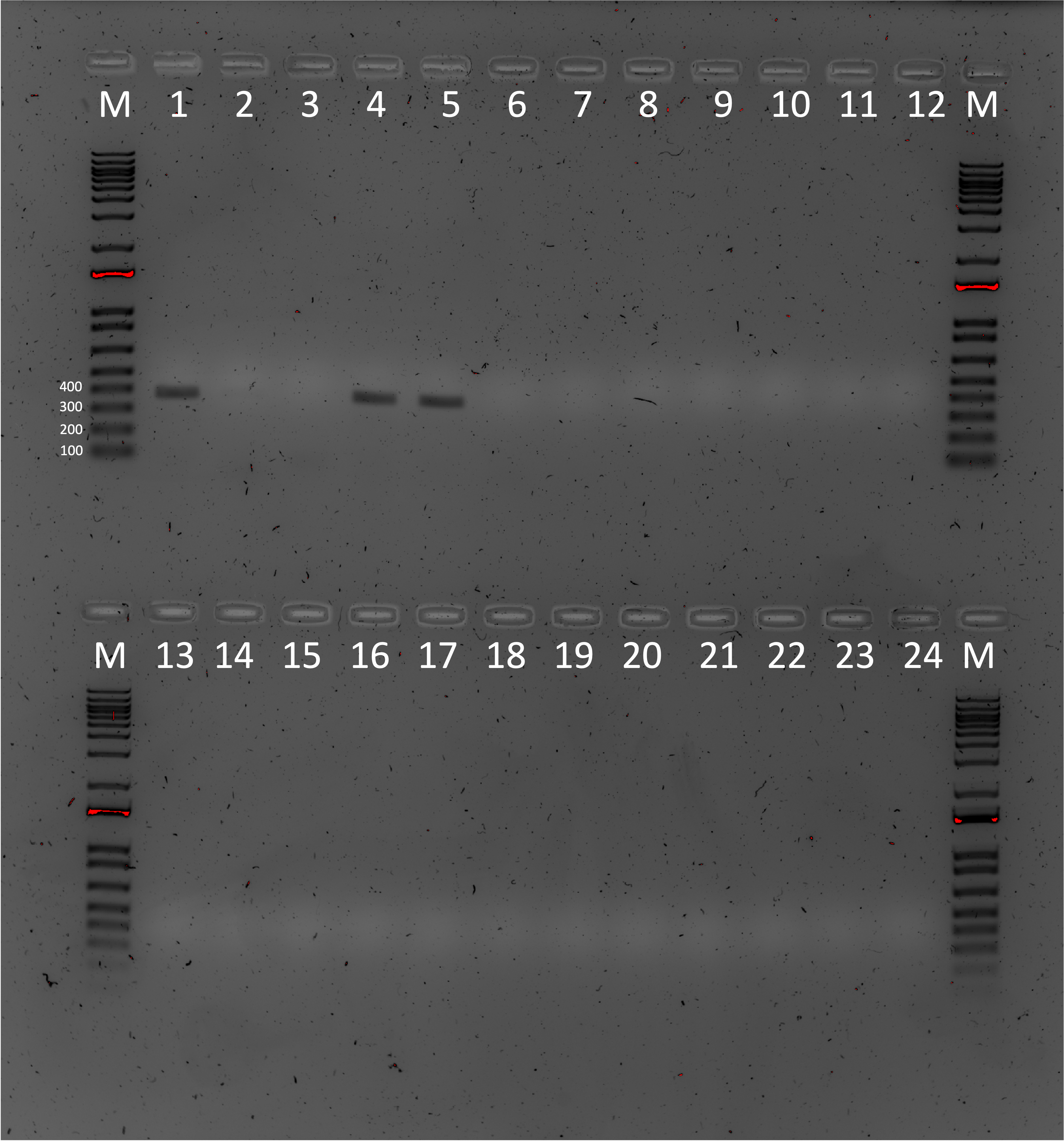

Supplement: S1 Fig — Results show that reactions using XDR primers with just the one SNP show amplification on non-XDR DNA samples. However, with the addition of a second SNP at the 3’ end of the primer, no amplification is present in the same non-XDR DNA samples. Lane M, 1kb DNA ladder; lanes 1, 7, 13 and 19 S. Typhi BRD948; lanes 2, 8, 14 and 20; S. Paratyphi A; lanes 3, 9, 15 and 21, S. Paratyphi B; lanes 4, 10, 16 and 22, S. Typhi H58 12148; lanes 5, 11, 17 and 23, S. Typhi H58 12960; lanes 6, 12, 18 and 24, No template control (NTC). (PNG) [file pone.0267805.s001.png]

Figure 3

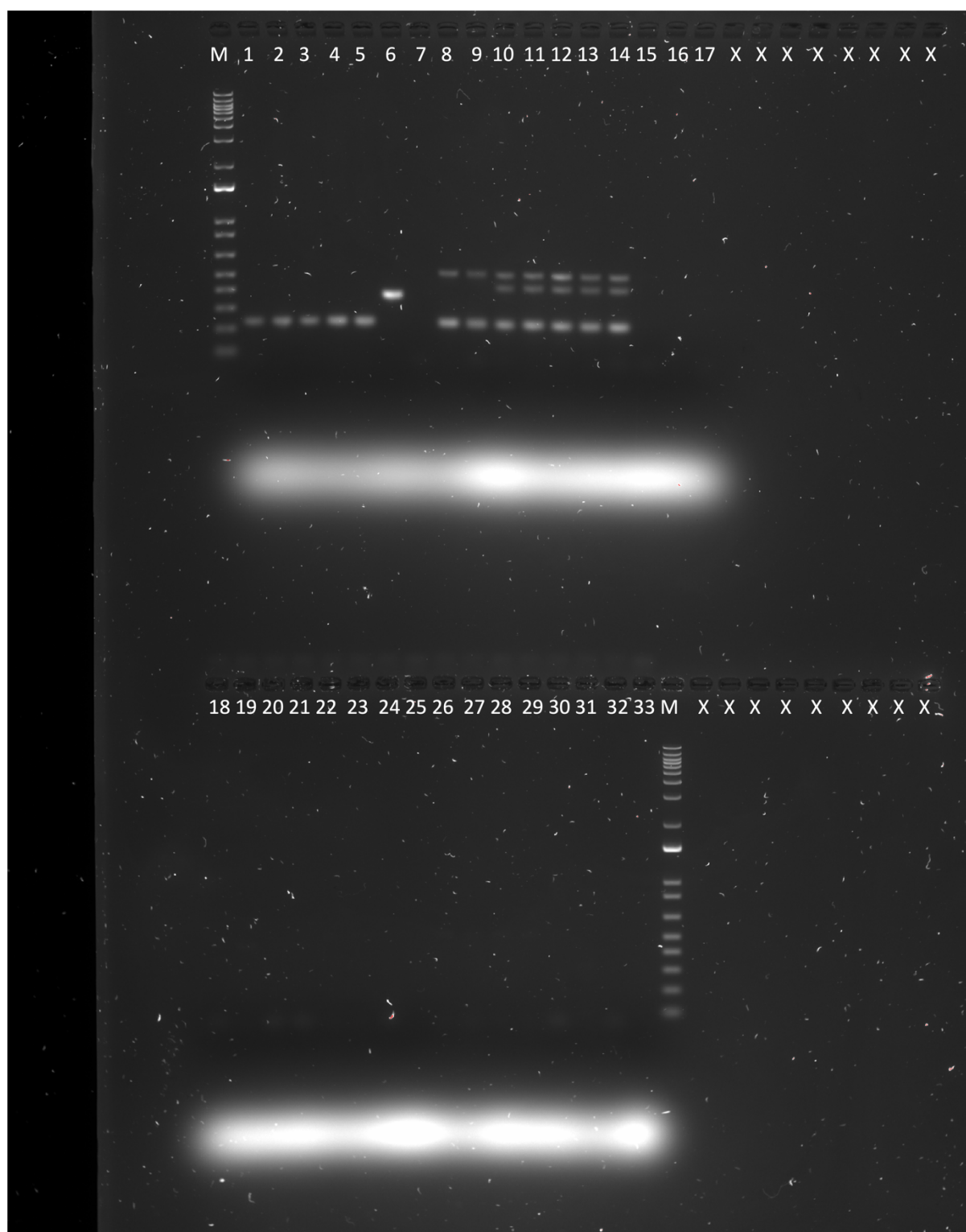

Supplement: S1 Raw images — (PDF) [file pone.0267805.s003.pdf]
