# Supplementary material for: Multiplex PCR assay to detect high risk lineages of Salmonella Typhi and Paratyphi A
Source: PLoS One. 2022 Jul 22;17(7):e0267805. doi: 10.1371/journal.pone.0267805 (PMC9307194; doi:10.1371/journal.pone.0267805)
Supplement: S1 Table — Samples identified as S. Paratyphi A with our PCR assay are highlighted in green. For these samples the result of subsequent result of whole genome sequencing is also listed. Samples that did not produce any bands are highlighted in yellow. (DOCX) [file pone.0267805.s002.docx]

| Lab ID | MALDI-TOF ID | PCR ID | WGS ID |
| --- | --- | --- | --- |
| S. Typhi | - | S. Typhi |  |
| S. Paratyphi A | - | S. Paratyphi A |  |
| S. Paratyphi B | - | no band |  |
| S. Typhi H58 | - | S. Typhi H58 |  |
| S. Typhi XDR | - | S. Typhi XDR |  |
| T1 | S. Typhi | S. Typhi H58 |  |
| T2 | S. Typhi | S. Typhi H58 |  |
| T3 | S. Typhi | S. Typhi H58 |  |
| T4 | S. Typhi | S. Paratyphi A | S. Paratyphi A |
| T5 | S. Paratyphi A | S. Paratyphi A | S. Paratyphi A |
| T6 | S. Typhi | S. Typhi H58 |  |
| T7 | S. Typhi | S. Typhi H58 |  |
| T8 | S. Typhi | S. Typhi H58 |  |
| T9 | S. Typhi | S. Typhi H58 |  |
| T10 | S. Typhi | S. Paratyphi A | S. Paratyphi A |
| T11 | S. Typhi | S. Paratyphi A | S. Paratyphi A |
| T12 | S. Typhi | S. Typhi H58 |  |
| T13 | S. Typhi | no band |  |
| T14 | S. Typhi | S. Typhi H58 |  |
| T15 | S. Typhi | S. Paratyphi A | S. Paratyphi A |
| T16 | S. Typhi | S. Typhi H58 |  |
| T17 | S. Typhi | S. Paratyphi A | S. Paratyphi A |
| T18 | S. Typhi | S. Typhi H58 |  |
| T19 | S. Typhi | S. Typhi H58 |  |
| T20 | S. Typhi | S. Typhi H58 |  |
| T21 | S. Typhi | S. Typhi H58 |  |
| T22 | S. Typhi | S. Typhi H58 |  |
| T23 | S. Typhi | S. Typhi H58 |  |
| T24 | S. Typhi | S. Typhi H58 |  |
| T25 | S. Typhi | S. Typhi H58 |  |
| T26 | S. Typhi | S. Typhi H58 |  |
| T27 | S. Typhi | S. Typhi H58 |  |
| T28 | S. Typhi | S. Typhi H58 |  |
| T29 | S. Typhi | S. Typhi H58 |  |
| T30 | S. Typhi | S. Typhi H58 |  |
| T31 | S. Typhi | S. Paratyphi A | S. Paratyphi A |
| T32 | S. Typhi | S. Typhi H58 |  |
| T33 | S. Typhi | S. Typhi H58 |  |
| T34 | S. Typhi | S. Typhi H58 |  |
| T35 | S. Typhi | no band |  |
| T36 | S. Typhi | S. Typhi H58 |  |
| T37 | S. Typhi | S. Typhi (faint) |  |
| T38 | S. Typhi | S. Paratyphi A | S. Paratyphi A |
| T39 | S. Typhi | S. Paratyphi A | S. Paratyphi A |
| T40 | S. Typhi | S. Typhi H58 |  |
| T41 | S. Typhi | S. Paratyphi A | S. Paratyphi A |
| T42 | S. Typhi | S. Typhi H58 |  |
| T43 | S. Typhi | S. Typhi H58 |  |
| T44 | S. Typhi | S. Typhi (faint) |  |
| T45 | S. Typhi | S. Typhi |  |
| T46 | S. Typhi | S. Typhi H58 |  |
| T47 | S. Typhi | S. Typhi H58 |  |
| T48 | S. Typhi | S. Paratyphi A | S. Paratyphi A |
| T49 | S. Typhi | S. Paratyphi A | S. Paratyphi A |
| T50 | S. Typhi | S. Typhi H58 |  |
| T51 | S. Typhi | S. Typhi H58 |  |
| T52 | S. Typhi | S. Typhi |  |
| T53 | S. Typhi | S. Typhi H58 |  |
| T54 | S. Typhi | S. Typhi H58 |  |
| T55 | S. Typhi | S. Typhi H58 |  |
| T56 | S. Typhi | S. Typhi H58 |  |
| T57 | S. Typhi | S. Typhi H58 |  |
| T58 | S. Typhi | S. Typhi H58 |  |
| T59 | S. Typhi | S. Typhi H58 |  |
| T60 | S. Typhi | S. Typhi H58 |  |
| T61 | S. Typhi | S. Typhi H58 |  |
| T62 | S. Typhi | S. Typhi and S. Paratyphi A |  |
| T63 | S. Typhi | S. Typhi H58 |  |
| T64 | S. Typhi | S. Paratyphi A | S. Paratyphi A |
| T65 | S. Typhi | S. Typhi H58 |  |
| T66 | S. Typhi | S. Typhi H58 |  |
| T67 | S. Typhi | S. Typhi H58 |  |
| T68 | S. Typhi | no band |  |
| T69 | S. Typhi | S. Typhi H58 |  |
| T70 | S. Typhi | S. Typhi H58 |  |
| T71 | S. Typhi | S. Typhi H58 |  |
| T72 | S. Typhi | S. Typhi H58 |  |
| T73 | S. Typhi | S. Typhi H58 |  |
| T74 | S. Typhi | S. Paratyphi A | S. Paratyphi A |
| T75 | S. Typhi | S. Typhi H58 |  |
